# Routine Electrocardiogram Screening and Cardiovascular Disease Events in Adults

**DOI:** 10.1001/jamainternmed.2024.2270

**Published:** 2024-07-01

**Authors:** Ryuichiro Yagi, Yuichiro Mori, Shinichi Goto, Taku Iwami, Kosuke Inoue

**Affiliations:** 1 Division of Cardiovascular Medicine, Department of Medicine, Brigham and Women’s Hospital, Boston, Massachusetts; 2 Harvard Medical School, Boston, Massachusetts; 3 Department of Human Health Sciences, Graduate School of Medicine, Kyoto University, Kyoto, Japan; 4 Division of General Internal Medicine & Family Medicine, Department of General and Acute Medicine, Tokai University School of Medicine, Isehara, Japan; 5 Department of Preventive Services, Graduate School of Medicine, Kyoto University, Kyoto, Kyoto, Japan.; 6 Department of Social Epidemiology, Graduate School of Medicine, Kyoto University, Kyoto, Kyoto, Japan; 7 Hakubi Center for Advanced Research, Kyoto University, Kyoto, Japan

## Abstract

**Importance:**

The resting electrocardiogram (ECG) is commonly performed for cardiovascular disease (CVD) screening purposes in Japan. However, evidence is limited regarding the prognostic significance of ECG in clinical practice settings.

**Objective:**

To investigate the association between ECG abnormalities and CVD outcomes in a working-age population.

**Design, Setting, and Participants:**

This nationwide cohort study included individuals aged 35 to 65 years from the Japan Health Insurance Association database, which covers approximately 40% (30 million) of the working-age population in Japan. Data from April 1, 2015, to March 31, 2022, were included, and analysis was conducted from October 1, 2022, to April 11, 2024.

**Exposures:**

Baseline ECG status (normal, 1 minor abnormality, ≥2 minor abnormalities, or major abnormality).

**Main Outcomes and Measures:**

The primary outcome was a composite of overall death and CVD hospital admission due to myocardial infarction, stroke, or heart failure. The secondary outcome was developing a new major ECG abnormality over the years of screening.

**Results:**

Of 3 698 429 individuals enrolled in the nationwide annual health check program (mean [SD] age, 47.1 [8.5] years; 66.6% male), 623 073 (16.8%) had 1 minor ECG abnormality, 144 535 (3.9%) had 2 or more minor ECG abnormalities, and 56 921 (1.5%) had a major ECG abnormality. During a median follow-up of 5.5 (IQR, 3.4-5.7) years, baseline ECG abnormality was independently associated with an increased incidence of the composite end points of overall death and CVD admission compared with normal ECG (incidence rates per 10 000 person-years: 92.7 [95% CI, 92.2-93.2] for normal ECG, 128.5 [95% CI, 127.2-129.9] for 1 minor ECG abnormality, 159.7 [95% CI, 156.6-162.9] for ≥2 minor ECG abnormalities, and 266.3 [95% CI, 259.9-272.3] for a major ECG abnormality; adjusted hazard ratios: 1.19 [95% CI, 1.18-1.20] for 1 minor ECG abnormality, 1.37 [95% CI, 1.34-1.39] for ≥2 minor ECG abnormalities, and 1.96 [95% CI, 1.92-2.02] for a major ECG abnormality). Furthermore, the presence and number of minor ECG abnormalities were associated with an increased incidence of developing new major ECG abnormalities (incidence rates per 10 000 person-years: 85.1 [95% CI, 84.5-85.5] for normal ECG, 217.2 [95% CI, 215.5-219.0] for 1 minor ECG abnormality, and 306.4 [95% CI, 302.1-310.7] for ≥2 minor ECG abnormalities; and adjusted hazard ratios: 2.52 [95% CI, 2.49-2.55] for 1 minor ECG abnormality and 3.61 [95% CI, 3.55-3.67] for ≥2 minor ECG abnormalities). Associations were noted regardless of baseline CVD risk.

**Conclusions and Relevance:**

The findings of this study suggest that the potential role of routine ECG screening for early prevention of CVD events, along with the optimal follow-up strategy, should be examined in future studies.

## Key Points

**Question:**

What is the evidence on the clinical utility of routine resting electrocardiogram (ECG) screening on cardiovascular risk assessment in the working-age population?

**Findings:**

In this nationwide cohort study of more than 3.5 million working-age Japanese adults enrolled in the nationwide annual health check program, baseline ECG findings were associated with the risk of cardiovascular events. Furthermore, the presence and the number of baseline minor ECG abnormalities were associated with developing new major ECG abnormalities.

**Meaning:**

The results of this study suggest that routine ECG screening may help identify individuals at high risk of developing cardiovascular events.

## Teaser

This cohort study examines the use of routine electrocardiogram (ECG) screening in identifying individuals at risk for cardiovascular disease.

## Introduction

Cardiovascular disease (CVD) is the leading cause of mortality in the US and globally.^1,2^ Particularly, 6.2 million people died of CVD between the ages of 30 and 70 years in 2019, and the rate of premature CVD mortality continues to rise, underscoring its substantial burden.^3,4^ Thus, identifying high-risk individuals is crucial for CVD prevention, as key drugs for properly managing atherosclerotic factors have been developed.^5,6,7^ The resting electrocardiogram (ECG) has been proposed for screening purposes in asymptomatic populations as it is the most widely used, accessible, and least invasive cardiovascular test. However, the ECG may not be cost-effective and may potentially increase an unnecessary testing and care cascade through incidental findings.^8^ Because of insufficient evidence on whether screening ECG can improve health outcomes, the US Preventive Services Task Force^9^ and the European Society of Cardiology^10^ do not recommend performing screening ECG for individuals at low risk of CVD.

According to the recent US Preventive Services Task Force guideline, “a considerable number of studies have reported hazard ratios [HRs] and other measures of association between ECG changes and cardiovascular outcomes.”^11^ More than 30 studies have assessed the association of ECG abnormalities with the risk of cardiovascular events.^12^ However, the evidence on the clinical utility of routine ECG in the general population remains insufficient because most of the existing literature consists of observational studies conducted on outdated, nongeneral populations^13,14,15,16,17,18,19^ and for limited ECG abnormalities or outcomes.^20,21,22,23,24,25,26,27^ Furthermore, since these studies assessed the association of 1 baseline ECG result with clinical outcomes, there is a lack of evidence regarding the association of minor ECG abnormalities with the development of major ECG abnormalities.

Therefore, using a large nationwide database from the health insurance association in Japan, we investigated the association between ECG results (ie, minor or major abnormalities) and incident CVD and the association between minor ECG abnormalities and risk of developing major ECG abnormalities. Although many countries have not introduced ECG as a tool for CVD screening, the annual ECG has been mandatory for working-age individuals in Japan since 1989.^28^ Thus, our goal is to provide evidence in the clinical setting regarding ECG screening in this unique context using sequential ECG results obtained from annual health screenings.

## Methods

### Data Source and Study Population

The institutional review board of Kyoto University approved all study procedures and waived informed consent for participants because all data used in the study were anonymized by a third-party data provider. All methods were conducted in accordance with the Helsinki Declaration.^29^ The study followed the Strengthening the Reporting of Observational Studies in Epidemiology (STROBE) reporting guideline.^30^

In this cohort study, we used the nationwide database with claims and annual health screening data from the Japan Health Insurance Association (JHIA) between April 1, 2015, and March 31, 2022. The JHIA is the largest public health insurer in Japan and provides health insurance to employees in small and medium-sized enterprises and their dependents, covering approximately 40% (30 million) of the working-age population. The details of Japan’s annual health screening program have been described elsewhere.^31^ Briefly, it is mandatory for public health insurers to provide working-age individuals with health screening every year, and particularly, ECG is required at the time of employment and for those who are aged 35 years or older. Although individuals aged 36 to 39 years can be exempted to undergo ECG with physicians’ agreement, more than 70% of them have an ECG even during those periods (age 35 years, 71.8%; age 36-39 years, 71.8%; and age 40 years; 73.2% in our data; the participation rate for health check-ups among JHIA-insured individuals was 53.8% in 2015).^32^

Every year, insurers are required to provide their employees with opportunities for health screenings with full subsidization. Employees who undergo screenings receive their results directly from the screening facilities and must report them to their insurers. Those diagnosed with major ECG abnormalities are advised by their insurers to seek further examinations, which are covered by their health insurance. While insurers are mandated to make the recommendation for the follow-up of detected abnormalities, visiting physicians (eg, primary care physicians, cardiologists) is optional for employees. During this process, physicians are not directly notified of these results. The health screening data include information on demographic characteristics, medical history, and laboratory results. Claims data were linked with the *International Statistical Classification of Diseases and Related Health Problems, Tenth Revision* (*ICD-10*) disease code.

We first identified individuals who were registered to the insurance database as the primary insurer in 2015. Of those, individuals who were aged 35 to 65 years and undertook a 12-lead resting ECG in 2016 were included in the study population. Individuals with baseline CVD, major ECG abnormalities in 2015, or missing covariates were excluded.

### ECG Analysis

The diagnosis from standard resting 12-lead ECGs recorded for each patient was obtained from the database. All ECGs were first read by vendor machines and checked by at least 1 physician. Electrocardiogram diagnosis was made based on the Minnesota Code for ECG classification. The final reports were made by physicians, and ECG abnormalities were divided into minor or major abnormalities according to the guideline from the Japan Society of Ningen-Dock, which is broadly used for screening settings in Japan (eTable 1 in Supplement 1).^33^ Participants were categorized into 4 groups based on the ECG diagnosis in 2016 (normal ECG, 1 minor ECG abnormality, ≥2 minor ECG abnormalities, and major ECG abnormality).

### Risk Evaluation and Stratification

Since it is reported that the Framingham Risk Score tended to overestimate the CVD risk for the Japanese population,^34^ the Suita score was used for the baseline CVD risk assessment. The details of the Suita score have been described elsewhere.^34^ Briefly, the Suita score was developed to estimate the 10-year risk of coronary heart disease for the Japanese population based on a large population-based study. This score includes age, sex, smoking status, diabetes, blood pressure, total cholesterol level, high-density lipoprotein cholesterol level, and chronic kidney disease status. Since it was reported that the absolute 10-year CVD risk was about 1% in individuals with a CVD score of 40 or less,^34^ those with a score of 40 or less were classified in the low-risk group and those with a score higher than 40 were classified into the moderate-high risk group.

### Outcome Ascertainment

Our main outcome was a composite of overall death and CVD hospital admission. Because the database did not include information on the cause of death, overall death was used as an alternative to cardiovascular death. Cardiovascular disease hospitalization was defined as admission due to stroke (*ICD-10* codes I60-I63), heart failure (*ICD-10* codes I50, I110, I130, or I132), or myocardial infarction (*ICD-10* codes I21 or I22). We included only cases with a confirmed CVD diagnosis and excluded suspected cases from the primary end point. Additionally, we evaluated the incidence of new major ECG abnormality in 2017-2021 in individuals with a normal ECG, 1 minor ECG abnormality, and 2 or more minor ECG abnormalities (ie, after excluding individuals presenting with major ECG abnormalities in 2016 at baseline). All participants were followed up until March 2022, when the outcome occurred, or when the participants left JHIA coverage (ie, retirement or change of job).

### Covariates

The following baseline variables were extracted from the database: age, sex, CVD history, comorbidities (hypertension, diabetes, and dyslipidemia), smoking status, medication use, and measurements and laboratory results in health screening. Individuals with more than 100 cigarettes per lifetime, smoking duration of more than 6 months, and the last smoking within a month were considered current smokers. Medication use (antihypertensive, antidiabetic, and lipid-lowering drugs) was self-reported. Measurements included systolic blood pressure and body mass index, which was calculated as weight in kilograms divided by height in meters squared. Laboratory results included fasting glucose level, low-density lipoprotein cholesterol level, high-density lipoprotein cholesterol level, and estimated glomerular filtration rate (eGFR), calculated using a creatinine formula modified for Japanese individuals: eGFR = 194 × serum creatinine^−1.094^ × age^−0.287^ × α (α = 0.739 for women and α = 1 for men).^35^

### Statistical Analysis

Data analysis was conducted from October 1, 2022, to April 11, 2024. To examine the association between baseline ECG diagnosis and the risk of CVD events, the cumulative incidences of the composite outcome across the ECG groups were compared using Kaplan-Meier curves and log-rank test. Subgroups were examined to assess the interaction between baseline ECG diagnosis and risk factors. Multivariable Cox proportional hazards regression models were constructed to adjust for multiple clinical covariates. In our main model, age, sex, body mass index, comorbidities (hypertension, diabetes, and dyslipidemia), systolic blood pressure, glucose level, low-density lipoprotein cholesterol level, and eGFR were included. The associations of baseline ECG diagnosis and CVD events were also assessed in subgroups by age (<50, ≥50 years), sex (male, female), and comorbidities (the presence or absence of hypertension, diabetes, and dyslipidemia). These analyses were performed in low and moderate-high CVD risk populations. As a sensitivity analysis, we additionally included information on medication use (antihypertensive, antidiabetic, and lipid-lowering drugs) in our main model. We also examined the association between ECG abnormalities and each outcome (ie, overall death and CVD admission) separately. The proportional hazards assumption was assessed using Schoenfeld residuals.^36^

To examine the association between minor ECG abnormality and newly developed major ECG abnormality, we compared the cumulative incidences of new major ECG abnormality across 3 groups (normal, 1 minor abnormality, and ≥2 minor abnormalities) after excluding those with a major ECG abnormality at baseline. The incidence of major ECG abnormalities at each year was calculated using the inverse probability weighting approach to account for loss to follow-up. Subgroup analyses were conducted by age, sex, and comorbidities in low and moderate-high CVD risk populations as was done in the primary analysis. In addition, we examined the association between each component of minor and major ECG abnormality vs normal ECG finding and the composite end point using multivariable Cox proportional hazards regression models.

To understand the follow-up examination rate by ECG status, we calculated the number of individuals who underwent transthoracic echocardiograms or catheterization procedures (ie, coronary angiography and percutaneous coronary intervention) within 1 year after baseline ECG. These data were obtained from the claims database and stratified by their baseline ECG findings.

All tests were 2-tailed, and *P* < .05 was considered statistically significant. All statistical analyses were performed using R, version 4.3.1 (R Project for Statistical Computing).

## Results

### Participant Characteristics

**Figure 1.**
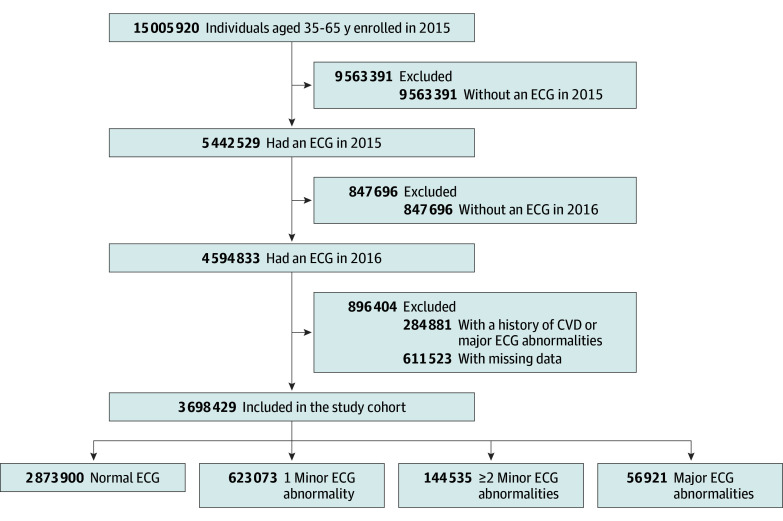
Flow of Participant Selection CVD indicates cardiovascular disease; ECG, electrocardiogram.

A total of 3 698 429 individuals who had ECGs performed in 2016 with no history of CVD or major ECG abnormalities in the previous year were included in our analyses (Figure 1). The mean (SD) age was 47.1 (8.5) years, 33.4% were female, and 66.6% were male (Table). The prevalence of hypertension was 14.1%; diabetes, 3.6%; and dyslipidemia, 8.1%. Normal ECG was found in 2 873 900 individuals (77.7%). One minor abnormality was observed in 623 703 individuals (16.8%), 144 535 (3.9%) had 2 or more minor abnormalities, and 56 921 (1.5%) had major ECG abnormalities. Individuals with a major ECG abnormality were older and had a higher prevalence of comorbidities compared with those without a major ECG abnormality. Nearly 90% of individuals with a normal ECG in 2015 did not show any ECG abnormality in 2016, and 40% of those with a major ECG abnormality in 2015 were found to have a major ECG abnormality in 2016 (eTable 2 in Supplement 1).

**Table.** Baseline Characteristics of the Study Population^a^

Characteristic	No. (%)				
	Overall (N = 3 698 429)	Normal ECG (n = 2 873 900 [77.7%])	1 Minor ECG abnormality (n = 623 073 [16.8%])	≥2 Minor ECG abnormalities (n = 144 535 [3.9%])	Major ECG abnormality (n = 56 921 [1.5%])
Age, mean (SD), y	47.1 (8.5)	46.8 (8.4)	48.0 (8.8)	48.8 (8.9)	49.8 (8.7)
Sex					
Male	2 463 899 (66.6)	1 886 808 (65.7)	434 988 (69.8)	102 664 (71.0)	39 439 (69.3)
Female	1 234 530 (33.4)	987 092 (34.3)	188 085 (30.2)	41 871 (29.0)	17 482 (30.7)
BMI, mean (SD)	23.4 (3.8)	23.4 (3.8)	23.2 (3.9)	23.1 (4.0)	23.7 (4.3)
Comorbidity					
Hypertension	521 843 (14.1)	379 964 (13.2)	102 570 (16.5)	26 603 (18.4)	12 706 (22.3)
Diabetes	132 379 (3.6)	224 360 (7.8)	55 943 (9.0)	13 950 (9.7)	6598 (11.6)
Dyslipidemia	300 851 (8.1)	97 680 (3.4)	25 105 (4.0)	6642 (4.6)	2952 (5.2)
Current smoker	1 272 550 (34.4)	985 191 (34.3)	216 432 (34.7)	50 372 (34.9)	20 555 (36.1)
Laboratory data					
SBP, mean (SD), mm Hg	122.3 (17.5)	121.8 (16.9)	123.7 (18.6)	124.7 (19.7)	129.2 (21.2)
Glucose, mean (SD), mg/dL	97.6 (20.1)	97.2 (19.5)	98.5 (21.2)	99.2 (22.5)	102.2 (26.7)
LDL-C, mean (SD), mg/dL	123.5 (31.6)	123.8 (31.6)	123.8 (31.6)	121.9 (31.6)	123.8 (32.9)
eGFR, mL/min/1.73 cm^2^	80.2 (14.5)	80.4 (14.4)	79.6 (14.7)	79.0 (15.2)	78.7 (15.9)
Medication					
Antihypertensive drugs	459 139 (12.4)	333 113 (11.6)	91 154 (14.6)	23 612 (16.3)	11 260 (19.8)
Antidiabetic drugs	138 137 (3.7)	103 168 (3.6)	25 515 (4.1)	6214 (4.3)	3240 (5.7)
Lipid-lowering drugs	265 234 (7.2)	201 068 (7.0)	47 819 (7.7)	11 050 (7.6)	5297 (9.3)
CVD risk score^b^					
Median (IQR)	36 (29-43)	36 (28-43)	36 (29-44)	37 (29-45)	40 (31-48)
>40	1 198 649 (33.0)	886 540 (31.4)	227 525 (37.3)	58 082 (40.8)	26 502 (47.6)
Composite event	169 093 (4.6)	118 210 (4.1)	34 781 (5.6)	9863 (6.8)	6239 (11.0)

Abbreviations: BMI, body mass index (calculated as weight in kilograms divided by height in meters squared); CVD, cardiovascular disease; ECG, electrocardiogram; eGFR, estimated glomerular filtration rate; LDL-C, low-density lipoprotein cholesterol; SBP, systolic blood pressure.

SI conversion factors: To convert glucose to millimoles per liter, multiply by 0.0555; LDL-C to millimoles per liter, multiply by 0.0259.

^a^ All differences significant at *P* < .001.

^b^ Suita score was used for the baseline CVD risk assessment.

### Association of Baseline ECG Diagnosis With CVD Events and New Major ECG Abnormality

**Figure 2.**
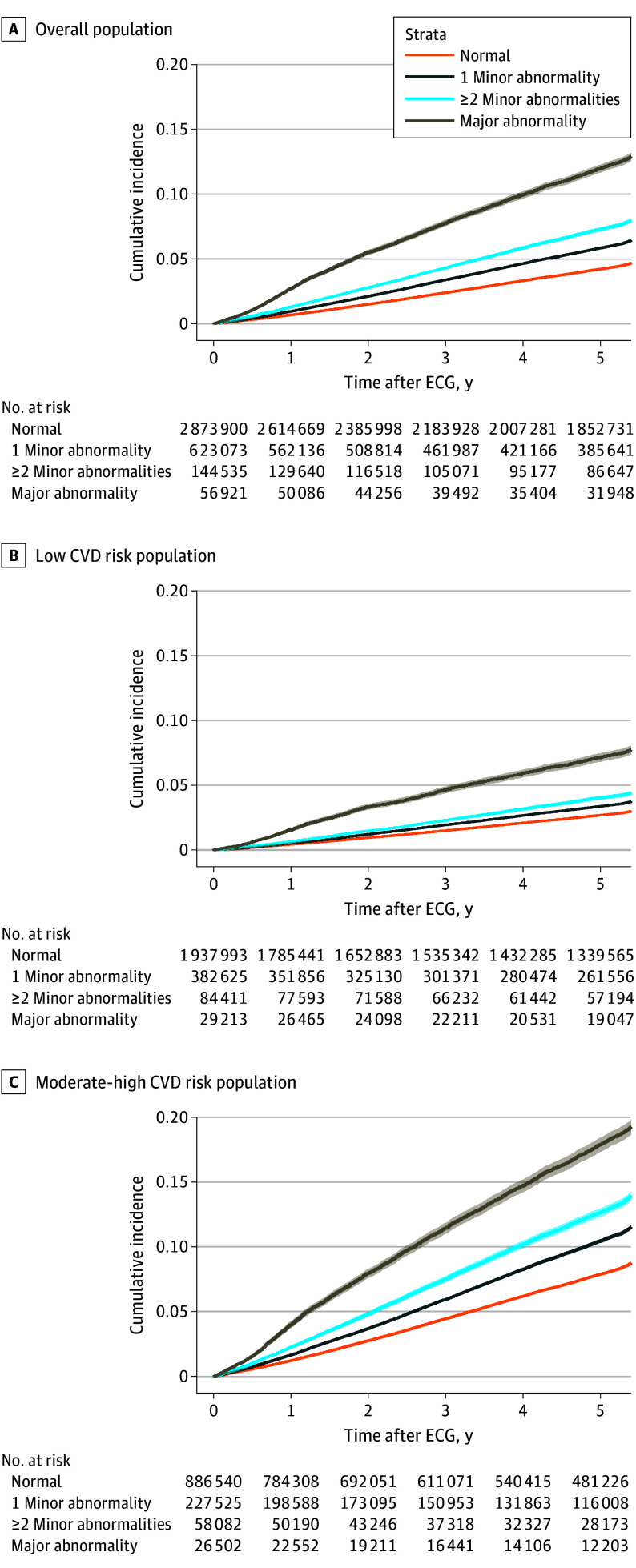
Cumulative Incidence of Composite End Point (Overall Death and Cardiovascular Disease [CVD] Admission) by Baseline Electrocardiogram (ECG) Status Cumulative incidence of composite end point across groups by baseline ECG status in overall population (A), low CVD risk population (B), and moderate-high CVD risk population (C).

During the median follow-up period of 5.5 (IQR, 3.4-5.7) years, 156 258 individuals (4.2%) experienced the composite end point. Baseline ECG abnormalities were associated with an increased incidence of the composite end point of overall death and CVD admission (χ^2^ = 11 122; log-rank *P* < .001) (Figure 2A). Incidence rates of the composite end point per 10 000 person-years were 103.7 (95% CI, 103.2-104.2) for the overall population, 92.7 (95% CI, 92.2-93.2) for a normal ECG, 128.5 (95% CI, 127.2-129.9) for 1 minor ECG abnormality, 159.7 (95% CI, 156.6-162.9) for 2 or more minor ECG abnormalities, and 266.3 (95% CI, 259.9-272.3) for a major ECG abnormality. This finding was significant in both low (χ^2^ = 2715; log-rank *P* < .001) and moderate-high (χ^2^ = 4810; log-rank *P* < .001) CVD risk groups when we stratified by the CVD risk score (Figure 2B and 2C). Cox proportional hazards regression model analysis showed individuals with baseline ECG abnormalities had a higher incidence of the composite outcome events compared with those with a normal ECG after adjusting for potential confounders (adjusted HR, 1.19 [95% CI, 1.18-1.20] for 1 minor ECG abnormality, 1.37 [95% CI, 1.34-1.39] for ≥2 minor ECG abnormalities, and 1.96 [95% CI, 1.92-2.02] for a major ECG abnormality). The results were consistent when additionally adjusted for information on medication use (eTable 3 in Supplement 1). We found similar results for both overall death and CVD admission when analyzing these outcomes separately (eTable 4 in Supplement 1). Similarly, individuals who did not have a history of CVD or ECG tests at baseline showed an incidence rate of 129.8 (95% CI, 129.5-130.1) per 10 000 person-years. The proportional hazards assumption was not violated in these analyses (eFigure 1 in Supplement 1).

**Figure 3.**
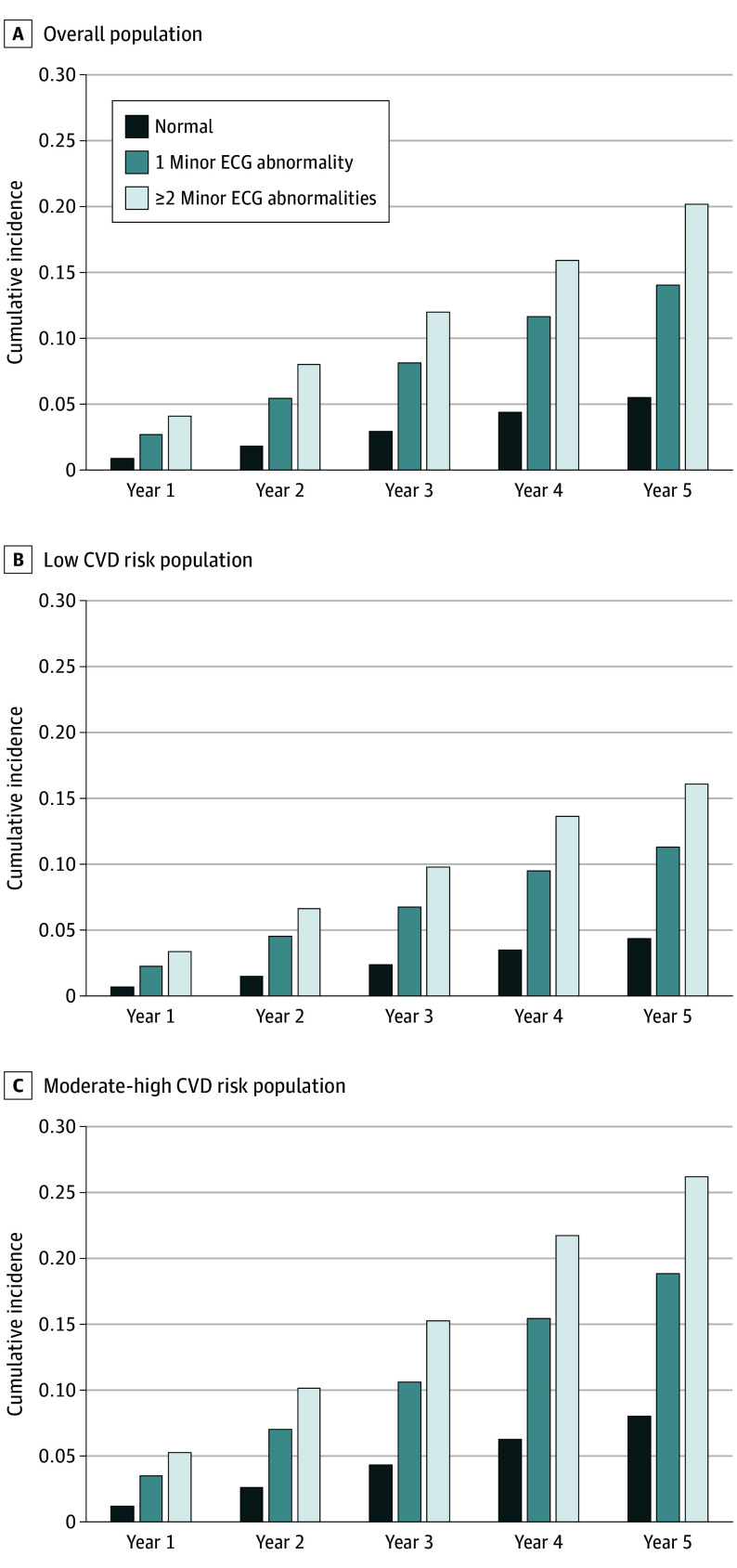
Cumulative Incidence of New Major Electrocardiogram (ECG) Abnormality by Baseline ECG Status Cumulative incidence of new major ECG abnormality across groups by baseline ECG status in overall population (A), low cardiovascular disease (CVD) risk population (B), and moderate-high CVD risk population (C).

Across a total of 3 698 429 individuals, 88.9% underwent ECGs in 2017, 79.4% in 2018, 72.7% in 2019, 62.5% in 2020, and 60.9% in 2021 (eTable 5 in Supplement 1). The proportion of follow-up ECGs tended to be slightly higher among individuals with a normal ECG than those with ECG abnormalities. Among those without a major ECG abnormality in 2016, 34 510 individuals (5.1%) developed at least 1 new major ECG abnormality during follow-up. The presence and number of minor ECG abnormalities were associated with an increased incidence of developing new major ECG abnormalities compared with normal ECG (Figure 3A). Incidence rates of major ECG abnormality per 10 000 person-years were 122.4 (95% CI, 121.9-122.9) for the overall population, 85.1 (95% CI, 84.5-85.5) for normal ECG, 217.2 (95% CI, 215.5-219.0) for 1 minor ECG abnormality, and 306.4 (95% CI, 302.1-310.7) for 2 or more minor ECG abnormalities. We found a similar pattern in the low and moderate-high CVD risk groups (Figure 3B and C). The multivariate Cox proportional hazards regression model demonstrated that individuals with minor ECG abnormalities were at high risk of developing a new major ECG abnormality after adjusting for potential confounders (adjusted HR, 2.52 [95% CI, 2.49-2.55] for 1 minor ECG abnormality and 3.61 [95% CI, 3.55-3.67] for ≥2 minor ECG abnormalities).

### Subgroup Analyses by Clinical Characteristics

**Figure 4.**
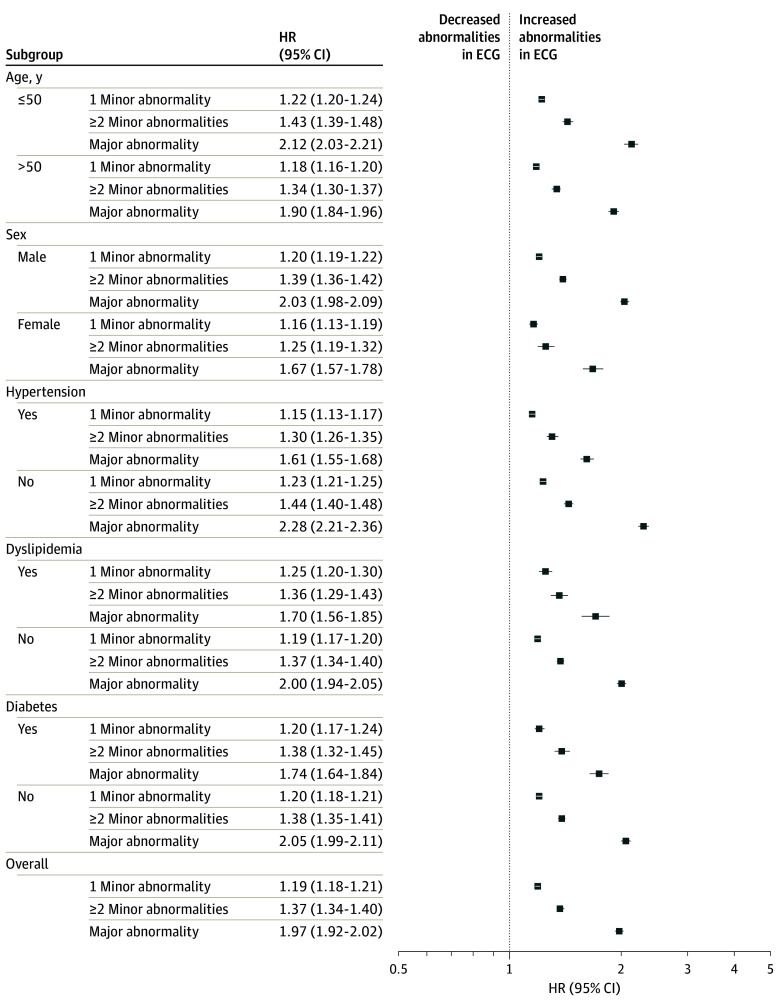
Comparison of the Risks of Composite End Point (Overall Death and Cardiovascular Disease Admission) Presented hazard ratios (HRs) for end point were adjusted for age, sex, body mass index, comorbidity (hypertension, diabetes, and dyslipidemia), systolic blood pressure, glucose level, low-density lipoprotein level, and estimated glomerular filtration rate. ECG indicates electrocardiogram.

Consistent results of the associations between baseline ECG abnormality and the risk of CVD events were observed across subgroups by age, sex, and presence or absence of clinical features among the overall population, low CVD risk groups, and moderate-high CVD risk groups (Figure 4; eFigure 2 in Supplement 1). Likewise, the associations between baseline ECG abnormality and the risk of developing a new major ECG abnormality were observed across subgroups by age, sex, and the presence or absence of clinical features regardless of baseline CVD risk score (eFigure 3 in Supplement 1).

### Association of Each ECG Abnormality and the Composite End Point

The multivariable Cox proportional hazards regression analyses showed that, across 29 types of minor abnormalities, 18 abnormalities were associated with an increased incidence of the composite end point (eg, HR for atrioventricular block [Wenckebach phenomenon], 2.06 [95% CI, 1.26-3.36]; high P wave amplitude, 1.91 [95% CI, 1.71-2.12]; high left ventricular voltage, 1.71 [95% CI, 1.67-1.75]; nonspecific ST-T wave change, 1.70 [95% CI, 1.63-1.77]; and abnormal T wave, 1.56 [95% CI, 1.52-1.60]) (eTable 6 in Supplement 1). Across 24 types of major ECG abnormalities, 19 abnormalities were associated with an increased incidence of the composite end point (eTable 7 in Supplement 1).

### Association of Baseline ECG Status and Follow-Up Diagnostics

Observed rates of follow-up echocardiography were 2.6% in individuals with normal ECG, 4.3% in individuals with 1 minor ECG abnormality, 5.6% in those with 2 or more minor ECG abnormalities, and 26.0% in individuals with a major ECG abnormality (eTable 8 in Supplement 1). Rates of follow-up coronary angiography were 0.2% in individuals with normal ECG, 0.3% in individuals with 1 minor ECG abnormality, 0.4% in those with 2 or more minor ECG abnormalities, and 1.2% in individuals with a major ECG abnormality.

## Discussion

Using a nationwide database that extensively covers more than 3.5 million working-age individuals in Japan, we identified a consistent association between a diverse array of ECG abnormalities and clinical outcomes in a more generalizable population. Additionally, we observed that the presence and number of minor ECG abnormalities were linked to the emergence of new major ECG abnormalities for which response is typically recommended by guidelines. These findings were observed in clinical circumstances where suboptimal follow-up strategies were taken.

The US Preventive Services Task Force does not recommend routine ECG in low-risk populations because of the lack of evidence on whether adding ECG screening to current CVD risk assessment tools leads to directly improved decision-making as well as potential for harm.^9^ However, the current evidence has included studies with major limitations regarding their study population or design.^37,38^ Although some studies were conducted at the population level, most were based on the Third National Health and Nutrition Examination Survey cohort (conducted in 1988-1994).^16,19,39^ Additionally, the number of participants included in these studies was limited (16 000 at most^40^), and most of those studies were performed in nongeneral, selected populations for limited purposes, including post hoc analyses of randomized clinical trials,^14^ studies of older populations,^13,15,17^ and studies focused on particular ECG changes^21,22,24,27^ or clinical outcomes.^20,23,25,26^ Thus, given the recent advancement of treatment and prevention strategies in CVD over the past 2 decades, updated evidence reflecting current practice has been warranted.

To address this issue, we used sequential health screening data of more than 3.5 million individuals in Japan. Since 1989, Japan has implemented its own health screening program for the working-age population to prevent worsening of employees’ health.^41^ In this screening program, ECG tests, in addition to physical examinations and blood sample tests, such as glucose levels, are provided for full-time employees aged 35 years and older every year for free, making the JHIA database unique. Based on this feature, we elucidated the association between ECG abnormalities and subsequent CVD outcomes suggesting the clinical significance of routine ECG screening in healthy, working-age populations. In Japan, individuals who are diagnosed with major ECG abnormalities during their annual health screenings are recommended by their insurers to visit clinics or hospitals for further examinations, which are covered by their insurance. Although this recommendation is mandatory for insurers, visiting physicians (eg, primary care physicians, cardiologists) is optional for employees. Our analysis observed that a considerable number of individuals with a major ECG abnormality did not undergo follow-up examinations despite the detection of a new major ECG abnormality.

Our findings highlight the potential role of baseline minor ECG abnormalities. We identified an association of the presence and number of minor ECG abnormalities with the risk of incident CVD and the development of new major ECG abnormalities over time in a more generalizable population, supported by previous studies performed in limited populations.^14,15,19^ Our analysis further enabled a comprehensive distinction between minor ECG abnormalities associated with clinical outcomes and those that were not. While some major ECG abnormalities, such as atrial fibrillation, have indications for further diagnostics and possibly treatment,^42^ minor ECG abnormalities have often been overlooked, and there is currently no guideline recommendation specifically addressing the management of multiple minor ECG abnormalities. Thus, our findings could shed new light on the clinical importance of minor ECG abnormalities incidentally detected in health screening settings.

The critical challenge persists on whether appropriate interventions following the identification of high-risk cases by ECG are available. Although our findings indicate an association between ECG results and CVD events, they do not imply that ECG results themselves or additional interventions based on the ECG results mitigate the outcomes of CVD. In addition, there is a possibility that the observed increased risk of all-cause mortality among individuals with ECG abnormalities could be partially due to overtreatment, such as potentially unnecessary invasive procedures. Furthermore, effective intervention is not necessarily available for most of the ECG abnormalities despite their association of higher risk for CVD. Considering the low follow-up rate even in the major ECG abnormality group, providing mandatory ECG tests without a suboptimal follow-up strategy may be inadequate. In this context, it is imperative to assess the effectiveness of workup strategies for individuals with ECG abnormalities. For instance, adopting a personalized approach that considers both baseline CVD risks and ECG findings, along with the optimal follow-up examinations (eg, active monitoring using ECG, Holter monitors, echocardiography, and wearable devices), could potentially maximize the utility of ECG findings, which should be the subject of future research.

### Limitations

There are several limitations in the study. First, a consensus regarding the classification of major and minor ECG abnormalities remains elusive. In fact, the classification of major and minor ECG abnormalities has varied across studies.^14,15,16,17,18^ We observed that multiple ECG abnormalities that are often considered minor were associated with CVD risk. Therefore, additional evaluation of the stratification of ECG abnormalities in risk assessment is warranted. Second, as we defined CVD outcomes based on *ICD-10* codes, we cannot rule out the possibility that outcome was misclassified (ie, patients with ECG abnormality are likely to obtain diagnostic codes of CVD, leading to bias away from the null). Third, we included all-cause death in our primary composite outcome because causes of death were not available in the database, which requires additional evidence focusing on CVD death. Fourth, the research may exhibit self-selection bias as the cohort consisted solely of individuals who have undergone annual health screening, potentially excluding lower socioeconomic groups who are less inclined to participate in such screenings. We observed a higher incidence rate for the primary outcome among individuals without ECG at baseline compared with our analytical samples. Fifth, because of the lack of data on follow-up examinations (eg, echocardiogram reports), the presence of complications, and other relevant medical notes, we could not assess the potential influence of ECG results on clinical decisions, indications for follow-up examinations, or the potential harm caused by ECGs. Sixth, our findings of the working-age population may not be generalizable to other demographic cohorts.

## Conclusions

In a nationwide cohort study of more than 3.5 million working adults in Japan, baseline ECG abnormalities were associated with an increased incidence of overall death and CVD-associated admission. They were independently associated with an increased incidence of developing a new major ECG abnormality. Further studies are needed to elucidate the role of routine ECG screening for early prevention of CVD events, along with optimal follow-up strategies for both major and minor ECG abnormalities.

## Notes

Supplement 1. **eTable 1.** The Classification System of ECG Abnormality **eTable 2.** ECG Categorizations in 2015 and 2016 **eTable 3.** Sensitivity Analysis Additionally Adjusting for Information on Medication Use **eTable 4.** Hazard Ratio for Overall Death and CVD Admission, According to ECG Status **eTable 5.** Proportion of Follow-Up ECG in Individuals With ECG in 2016 **eTable 6.** Association of Each Minor ECG Abnormality With the Composite End Point **eTable 7.** Association of Each Major ECG Abnormality With the Composite End Point **eTable 8.** Number of Echocardiogram and Catheterization Procedures Within 1 Year After Baseline ECG **eFigure 1.** Schoenfeld Residual Plots **eFigure 2.** Comparison of the Risks of Composite End Point (Overall Death and CVD Admission) by Baseline ECG Status Across Subgroups by Baseline CVD Risk **eFigure 3.** Comparison of the Risks of New Major ECG Abnormality Across Subgroups by Baseline CVD Risk

Supplement 2.
Data Sharing Statement
