# Supplementary material for: Routine Electrocardiogram Screening and Cardiovascular Disease Events in Adults
Source: JAMA Intern Med. 2024 Jul 1;184(9):1035–44. doi: 10.1001/jamainternmed.2024.2270 (PMC11217891; doi:10.1001/jamainternmed.2024.2270)
Supplement: Supplement 1. — eTable 1. The Classification System of ECG Abnormality eTable 2. ECG Categorizations in 2015 and 2016 eTable 3. Sensitivity Analysis Additionally Adjusting for Information on Medication Use eTable 4. Hazard Ratio for Overall Death and CVD Admission, According to ECG Status eTable 5. Proportion of Follow-Up ECG in Individuals With ECG in 2016 eTable 6. Association of Each Minor ECG Abnormality With the Composite End Point eTable 7. Association of Each Major ECG Abnormality With the Composite End Point eTable 8. Number of Echocardiogram and Catheterization Procedures Within 1 Year After Baseline ECG eFigure 1. Schoenfeld Residual Plots eFigure 2. Comparison of the Risks of Composite End Point (Overall Death and CVD Admission) by Baseline ECG Status Across Subgroups by Baseline CVD Risk eFigure 3. Comparison of the Risks of New Major ECG Abnormality Across Subgroups by Baseline CVD Risk [file jamainternmed-e242270-s001.pdf]

Supplementary Online Content

Yagi R, Mori Y, Goto S, Iwami T, Inoue T. Routine electrocardiogram screening and cardiovascular disease events in adults. *JAMA Intern Med*. Published online July 1, 2024. doi:10.1001/jamainternmed.2024.2270

eTable 1. The Classification System of ECG Abnormality
eTable 2. ECG Categorizations in 2015 and 2016
eTable 3. Sensitivity Analysis Additionally Adjusting for Information on Medication Use
eTable 4. Hazard Ratio for Overall Death and CVD Admission, According to ECG Status
eTable 5. Proportion of Follow-Up ECG in Individuals With ECG in 2016
eTable 6. Association of Each Minor ECG Abnormality With the Composite End Point
eTable 7. Association of Each Major ECG Abnormality With the Composite End Point
eTable 8. Number of Echocardiogram and Catheterization Procedures Within 1 Year After Baseline ECG
eFigure 1. Schoenfeld Residual Plots
eFigure 2. Comparison of the Risks of Composite End Point (Overall Death and CVD Admission) by Baseline ECG Status Across Subgroups by Baseline CVD Risk
eFigure 3. Comparison of the Risks of New Major ECG Abnormality Across Subgroups by Baseline CVD Risk

This supplementary material has been provided by the authors to give readers additional information about their work.

© 2024 Yagi et al. *JAMA Internal Medicine*.

eTable 1. The classification system of ECG abnormality

Categorization	Diagnosis	Minnesota code
**Minor ECG abnormality**	A-V block (Wenckebach's phenomenon)	6-2-3
	Abnormal T wave	5-2~4
	Borderline Q wave	1-2-1~7, 1-3-4
	Complete RBBB	7-2-1
	Early depolarization	9-2-1
	High amplitude R wave: right	3-2
	High left ventricular voltage (without ST-T change)	3-1,3-3
	High P wave amplitude	9-3
	High T wave amplitude	9-5
	Incomplete RBBB	7-3
	Indetermined axis deviation	2-5
	Intermittent aberrant A-V conduction	6-6
	Intermittent RBBB	7-2-2
	Left anterior hemiblock (LAH)	7-7
	Left/extreme QRS axis deviation	2-1, 4
	Low QRS amplitude	9-1
	P-R interval ≥ 0.22 sec	6-3
	Persistent supraventricular rhythm	8-4-1
	Premature atrial contraction	8-1-1, 8-9-1
	Premature ventricular contraction	8-1-2
	R-R' pattern	7-5
	Right axis deviation	2-2,3
	Short PQ interval	6-5
	Sinus arrhythmia	8-9-2
	Sinus bradycardia (40-44 bpm)	8-8
	Sinus tachycardia (86-100 bpm)	8-7
	ST elevation	9-2
	Non-specific ST-T change	4-4
	Wandering atrial pacemaker	8-1-4
**Major abnormality**	A-V dissociation	8-6
	Abnormal Q wave	1-1
	Atrial fibrillation	8-3-1
	Atrial flutter	8-3-2
	Brugada type ST elevation	9-2-3,9-2-4
	Combination of LAH and complete RBBB	7-8
	Complete A-V block	6-1
	High amplitude R waves (biventricular)	3-4
	High left ventricular voltage (with ST-T change)	3-1, 3-3
	Intraventricular block	7-4
	Left bundle branch brock	7-1-1, 2
	Mobitz Type II A-V block	6-2-1
	Multifocal premature atrial contraction	8-1-1, 8-9-1
	Multifocal premature ventricular contraction	8-1-2
	Negative T wave (≥0.5mV)	5-1
	Partial A-V block (2:1)	6-2-2
	Poor R progression	1-2-8
	S-A block	8-5
	Sinus bradycardia (≤39 bpm)	8-8
	Sinus tachycardia (≥101 bpm)	8-7
	ST-T depression (horizontal/downsloping)	4-1~3
	Supraventricular tachycardia	8-4-2
	Ventricular tachycardia	8-2-1~3
	Wolf-Parkinson-White syndrome	6-4

ECG, electrocardiogram; A-V, atrioventricular; S-A, sinoatrial; RBBB, right bundle branch block

© 2024 Yagi et al. *JAMA Internal Medicine*.

eTable 2. ECG categorizations in 2015 and 2016

2016 / 2015	Total	Normal	1 minor ECG abnormality	≥2 minor ECG abnormalities	Major ECG abnormality
Normal	3476674	3077504 (88.5%)	315366 (9.1%)	48886 (1.4%)	34918 (1.0%)
1 minor ECG abnormality	715220	289968 (40.5%)	354797 (49.6%)	46337 (6.5%)	24118 (3.3%)
≥2 minor ECG abnormalities	166070	43414 (26.1%)	42304 (25.5%)	72103 (43.4%)	8249 (5.0%)
Major ECG abnormality	106929	31800 (29.4%)	23975 (22.4%)	8330 (7.8%)	42824 (40.0%)

ECG, electrocardiogram.

© 2024 Yagi et al. *JAMA Internal Medicine*.

eTable 3. Sensitivity analysis additionally adjusting for information on medication use

	Hazard ratio (95% confidence interval)
**A) Overall population**	
One minor ECG abnormality	1.19 [1.18-1.21]
≥2 minor ECG abnormalities	1.37 [1.34-1.39]
Major ECG abnormality	1.97 [1.92-2.02]
**B) Low CVD risk population**	
One minor ECG abnormality	1.18 [1.16-1.21]
≥2 minor ECG abnormalities	1.35 [1.30-1.40]
Major ECG abnormality	2.09 [2.00-2.18]
**C) Moderate-high CVD risk population**	
One minor ECG abnormality	1.21 [1.19-1.23]
≥2 minor ECG abnormalities	1.40 [1.36-1.43]
Major ECG abnormality	1.93 [1.87-1.99]

Presented HRs for endpoint were adjusted for age, sex, body mass index, comorbidity (hypertension, diabetes, and dyslipidemia), systolic blood pressure, glucose, low-density lipoprotein, estimated glomerular filtration rate, and medication use (antihypertensive drugs, antidiabetic drugs, and lipid-lowering drugs).

CVD, cardiovascular disease; ECG, electrocardiogram; HR, hazard ratio; CI, confidence interval

© 2024 Yagi et al. *JAMA Internal Medicine*.

eTable 4. Hazard ratio for overall death and CVD admission, according to ECG status

	Hazard ratio (95% confidence interval)		
	**Primary composite outcomes**	**Overall death**	**CVD admission**
**A) Overall population**			
One minor ECG abnormality	1.19 [1.18–1.20]	1.18 [1.14–1.23]	1.20 [1.18–1.21]
≥2 minor ECG abnormalities	1.37 [1.34–1.39]	1.37 [1.29–1.46]	1.36 [1.33–1.39]
Major ECG abnormality	1.96 [1.92–2.02]	1.83 [1.69–1.98]	1.90 [1.85–1.95]
**B) Low CVD risk population**			
One minor ECG abnormality	1.19 [1.17–1.21]	1.16 [1.10–1.23]	1.19 [1.17–1.22]
≥2 minor ECG abnormalities	1.35 [1.30–1.39]	1.29 [1.16–1.43]	1.36 [1.31–1.41]
Major ECG abnormality	2.05 [1.96–2.14]	1.64 [1.42–1.90]	2.10 [2.00–2.20]
**C) Moderate-high CVD risk population**			
One minor ECG abnormality	1.20 [1.18–1.22]	1.21 [1.16–1.27]	1.20 [1.18–1.22]
≥2 minor ECG abnormalities	1.38 [1.35–1.42]	1.45 [1.34–1.56]	1.38 [1.34–1.42]
Major ECG abnormality	1.84 [1.78–1.90]	1.93 [1.75–2.12]	1.82 [1.76–1.88]

Presented HRs for endpoint were adjusted for age, sex, body mass index, comorbidity (hypertension, diabetes, and dyslipidemia), systolic blood pressure, glucose, low-density lipoprotein, and estimated glomerular filtration rate.
CVD, cardiovascular disease; ECG, electrocardiogram; HR, hazard ratio; CI, confidence interval

© 2024 Yagi et al. *JAMA Internal Medicine*.

eTable 5. Proportion of follow–up ECG in individuals with ECG in 2016

ECG categorization	2016	2017	2018	2019	2020	2021
Normal	2873900	2560219 (89.1%)	2290656 (79.7%)	2099587 (73.1%)	1811487 (63.0%)	1766175 (61.5%)
1 minor ECG abnormality	623073	552432 (88.7%)	489869 (78.6%)	446371 (71.6%)	381258 (61.2%)	369834 (58.3%)
≥2 minor ECG abnormalities	144555	126905 (87.8%)	112836 (78.1%)	102390 (70.8%)	87200 (60.3%)	84226 (58.3%)
Major ECG abnormality	56921	49673 (87.3%)	43603 (76.6%)	39345 (69.1%)	33185 (58.3%)	31995 (56.2%)
Overall	3698448	3289229 (88.9%)	2936964 (79.4%)	2687693 (72.7%)	2313130 (62.5%)	2252230 (60.9%)

ECG, electrocardiogram.

© 2024 Yagi et al. *JAMA Internal Medicine*.

eTable 6. Association of each minor ECG abnormality with the composite end point

ECG abnormality	N	IR	95%CI	HR	95%CI	P [a]
Any minor ECG abnormality	767608	101	100 – 102	1.26	1.25 – 1.27	<0.01
A-V block (Wenckebach's phenomenon)	154	230	137 – 379	2.06	1.26 – 3.36	<0.01
Abnormal T wave	72245	215	210 – 220	1.56	1.52 – 1.60	<0.01
Borderline Q wave	4640	185	167 – 205	1.25	1.13 – 1.38	<0.01
Complete RBBB	53738	173	167 – 178	1.21	1.17 – 1.25	<0.01
Early depolarization	10022	72	65 – 80	0.91	0.82 – 1.02	0.13
High R wave amplitude: right	2187	83	67 – 104	1.12	0.90 – 1.39	0.38
High left ventricular voltage (without ST-T change)	77189	203	198 – 208	1.71	1.67 – 1.75	<0.01
High P wave amplitude	4447	180	161 – 201	1.91	1.71 – 2.12	<0.01
High T wave amplitude	11241	89	82 – 98	1.18	1.07 – 1.29	<0.01
Incomplete RBBB	56332	94	91 – 99	1.08	1.04 – 1.13	<0.01
Indetermined axis deviation	2584	80	64 – 98	1.02	0.83 – 1.25	0.87
Intermittent aberrant A-V conduction	12855	110	102 – 119	1.12	1.03 – 1.21	<0.01
Intermittent RBBB	41	177	46 – 550	1.43	0.46 – 4.43	0.62
Left anterior hemiblock	6660	168	153 – 184	1.34	1.22 – 1.47	<0.01
Left/extreme QRS axis deviation	120179	132	129 – 135	1.08	1.05 – 1.11	<0.01
Low QRS amplitude	27256	81	76 – 86	0.97	0.91 – 1.04	0.47
P-R interval ≥ 0.22 sec	25579	215	206 – 223	1.11	1.06 – 1.17	<0.01
Persistent supraventricular rhythm	608	163	156 – 171	1.14	0.75 – 1.73	0.6
Premature atrial contraction	20622	83	67 – 104	1.45	1.38 – 1.53	<0.01
Premature ventricular contraction	22542	182	173 – 191	1.36	1.30 – 1.43	<0.01
R-R' pattern	29215	167	162 – 178	1.01	0.96 – 1.07	0.69
Right axis deviation	32451	91	86 – 96	0.96	0.90 – 1.02	0.24
Short PQ interval	20093	61	57 – 65	1.14	1.05 – 1.23	<0.01
Sinus arrhythmia	32269	70	65 – 74	1.02	0.96 – 1.08	0.09
Sinus bradycardia (40-44 bpm)	87872	76	72 – 81	0.91	0.88 – 0.94	<0.01
Sinus tachycardia (86-100 bpm)	13758	79	77 – 82	1.54	1.45 – 1.63	<0.01
ST elevation	17076	197	186 – 208	1.11	1.03 – 1.20	<0.01
Nonspecific ST-T change	28025	94	87 –101	1.7	1.63 – 1.77	<0.01
Wandering atrial pacemaker	2993	70	57 – 87	0.98	0.80 – 1.20	0.85

ECG, electrocardiogram; IR, incidence rate (per 10,000 person-year); HR, hazard ratio; CI, confidence interval; A-V, atrioventricular; S-A, sinoatrial; RBBB, right bundle branch block; bpm, beat per minute

[a]P values were adjusted for multiple comparisons using the Benjamini-Hochberg method.

© 2024 Yagi et al. *JAMA Internal Medicine*.

eTable 7. Association of each major ECG abnormality with the composite end point

ECG abnormality	N	IR	95%CI	HR	95%CI	P [a]
Any major ECG abnormality	56921	266	260 – 273	1.97	1.92 – 2.02	<0.01
A-V dissociation	37	182	38 – 531	2.17	0.70 – 6.73	0.20
Abnormal Q wave	5745	335	312 – 360	2.16	2.01 – 2.31	<0.01
Atrial fibrillation	1943	1210	1130 – 1296	6.49	6.03 – 6.98	<0.01
Atrial flutter	103	1831	1401 – 2353	10.5	7.83 – 14.0	<0.01
Brugada type ST elevation	5869	94	83 – 106	1.23	1.08 – 1.39	<0.01
Combination of LAH and complete RBBB	55	290	128 – 614	2.78	1.33 – 5.83	<0.01
Complete A-V block	63	190	70 – 464	1.75	0.66 – 4.68	0.25
High amplitude R wave (biventricular)	1590	307	268 – 353	2.36	2.06 – 2.71	<0.01
High left ventricular voltage (with ST-T change)	4862	390	363 – 418	2.73	2.54 – 2.93	<0.01
Left bundle branch block	1092	336	286 – 394	2.29	1.95 – 2.69	<0.01
Morbitz type II A-V block	22	240	42 – 920	1.83	0.46 – 7.34	0.39
Multifocal PAC	1199	171	138 – 213	1.39	1.12 – 1.72	<0.01
Multifocal PVC	2196	205	177 – 236	1.62	1.40 – 1.87	<0.01
Negative T wave (≥0.5mV)	5980	94	83 – 107	1.57	1.53 – 1.62	<0.01
Partial A-V block (2:1)	5	648	34 – 3324	6.90	0.97 – 48.9	0.06
Poor R progression	5230	112	99 – 127	1.43	1.26 – 1.61	<0.01
S-A block	134	210	114 – 375	1.70	0.97 – 3.00	0.08
Sinus bradycardia (≤39 bpm)	2269	125	104 – 149	1.25	1.05 – 1.49	<0.01
Sinus tachycardia (≥101 bpm)	1106	313	264 – 369	4.26	2.61 – 6.96	<0.01
ST depression (horizontal/downsloping)	13289	272	259 – 286	2.05	1.95 – 2.16	<0.01
Supraventricular tachycardia	68	613	366 – 995	4.26	2.61 – 6.96	<0.01
Ventricular tachycardia	8	1410	371 – 3695	7.84	2.53 – 24.3	<0.01
Wolf-Parkinson-White syndrome	4148	204	185 – 226	2.40	2.17 – 2.66	<0.01

ECG, electrocardiogram; IR, incidence rate (per 10,000 person-year); HR, hazard ratio; CI, confidence interval; LAH, left anterior hemiblock; PAC, premature atrial contractions; PVC, premature ventricular contractions; A-V, atrioventricular; S-A, sinoatrial; RBBB, right bundle branch block; bpm, beat per minute
[a]P values were adjusted for multiple comparisons using the Benjamini-Hochberg method.

© 2024 Yagi et al. *JAMA Internal Medicine*.

eTable 8. Number of echocardiogram and catheterization procedures within 1 year after baseline ECG

ECG finding	N	N of transthoracic echocardiogram (%)	N of catheterization procedure (%)
Normal ECG	2873900	74482 (2.6)	4172 (0.15)
1 minor ECG abnormality	623073	26869 (4.3)	1693 (0.27)
≥ 2 minor ECG abnormalities	144535	8188 (5.7)	571 (0.40)
Major ECG abnormality	56921	14772 (26.0)	681 (1.20)
Overall	3698429	124331 (3.4)	7117 (0.19)

ECG; electrocardiogram.

eFigure 1. Schoenfeld residual plots

A. Overall population

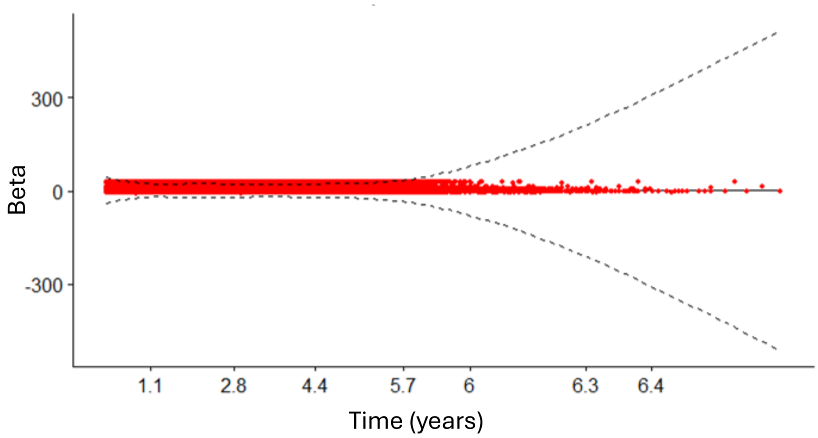

B. Low CVD risk population

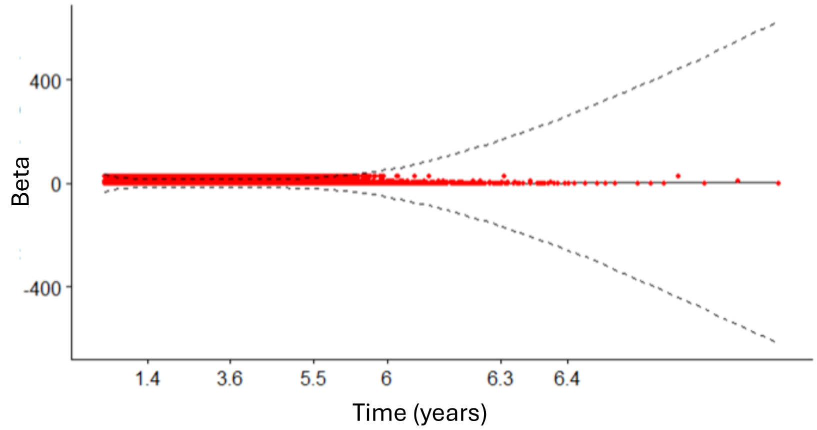

C. Moderate-high CVD risk population

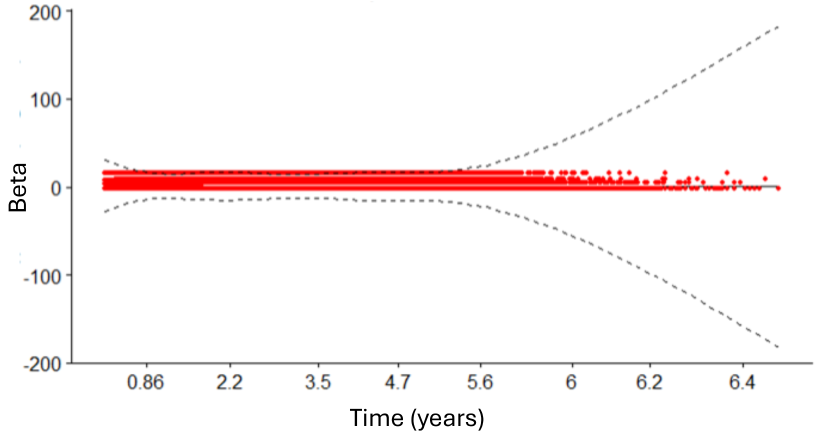

© 2024 Yagi et al. *JAMA Internal Medicine*.

eFigure 2. Comparison of the risks of composite endpoint (overall death and CVD admission) by baseline ECG status across subgroups by baseline CVD risk

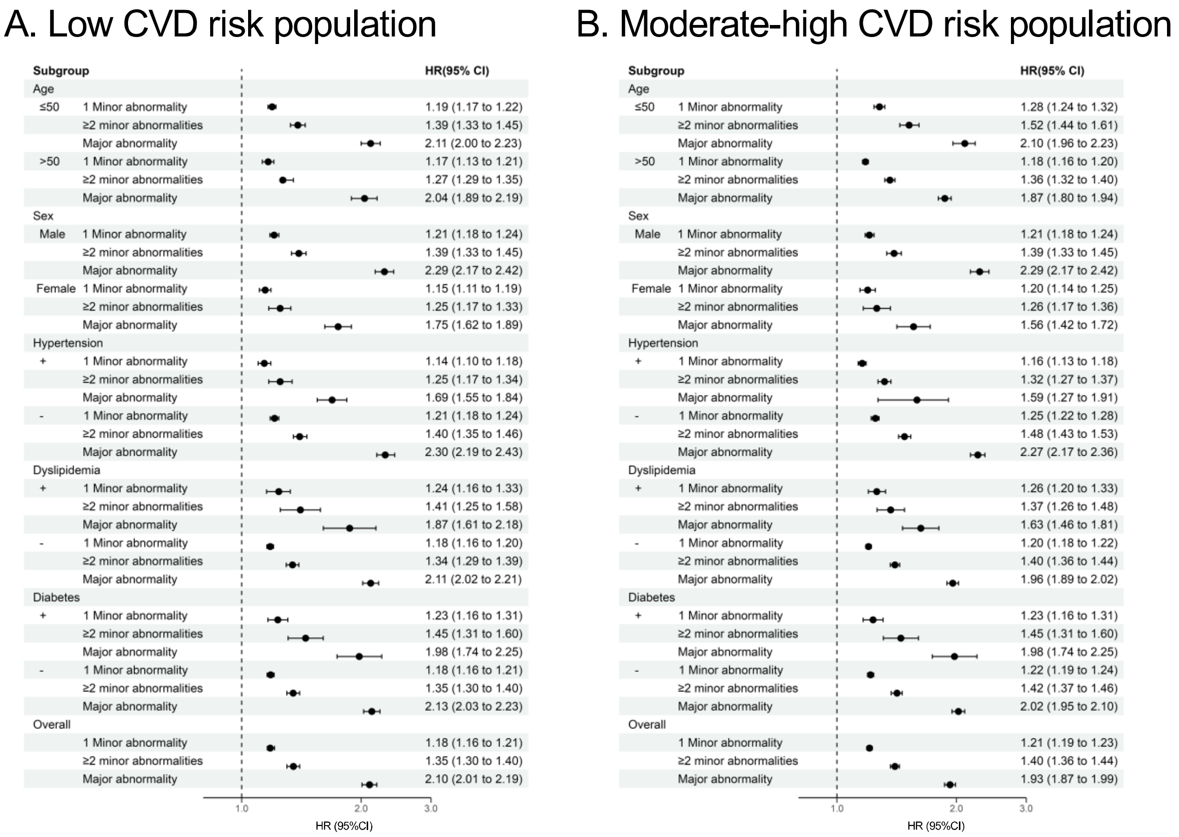

Presented HRs for endpoint were adjusted for age, sex, body mass index, comorbidity (hypertension, diabetes, and dyslipidemia), systolic blood pressure, glucose, low-density lipoprotein, and estimated glomerular filtration rate.

CVD, cardiovascular disease; ECG, electrocardiogram; HR, hazard ratio; CI, confidence interval

© 2024 Yagi et al. *JAMA Internal Medicine*.

eFigure 3. Comparison of the risks of new major ECG abnormality across subgroups by baseline CVD risk

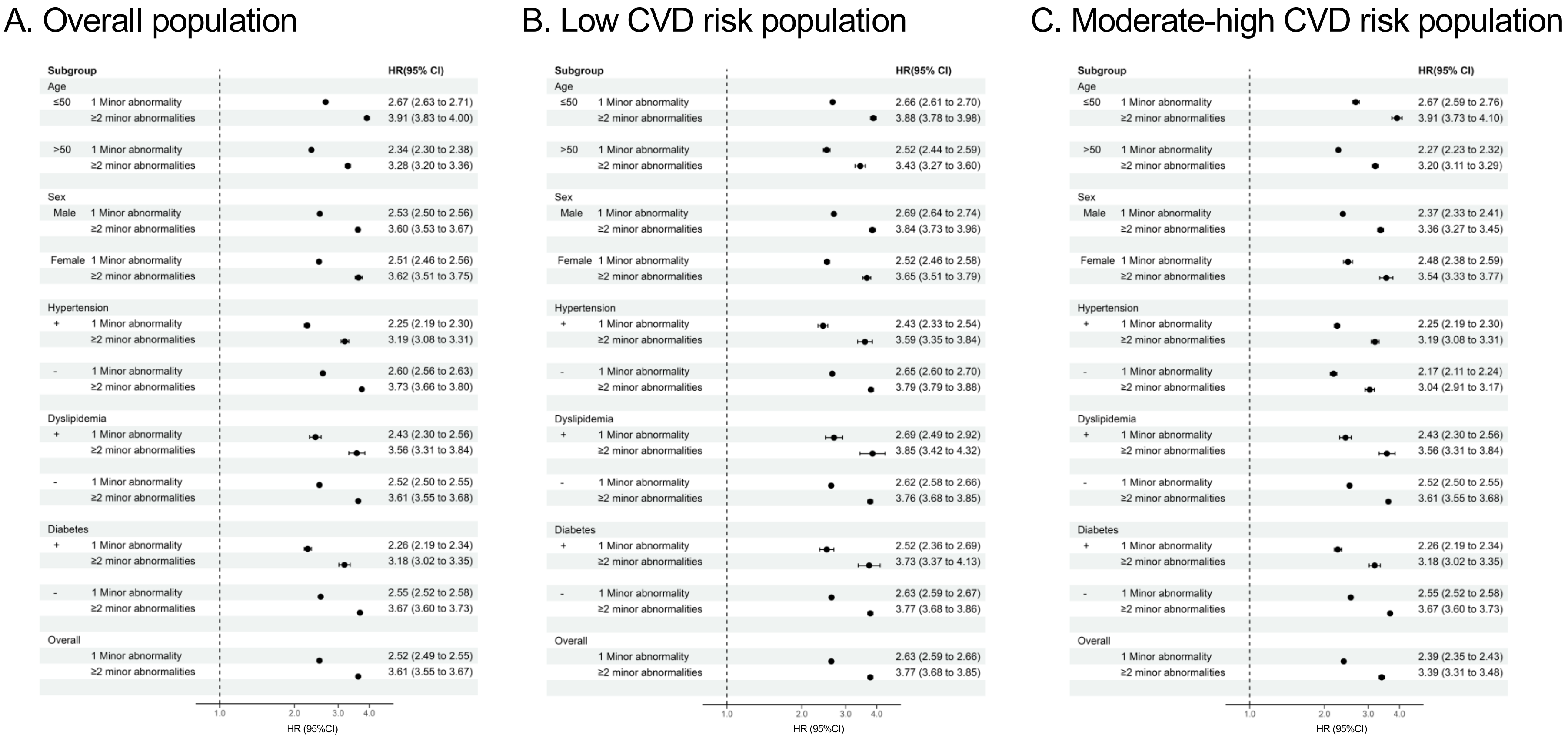

Presented HRs for new major ECG abnormality were adjusted for age, sex, body mass index, comorbidity (hypertension, diabetes, and dyslipidemia), systolic blood pressure, glucose, low-density lipoprotein, and estimated glomerular filtration rate.

CVD, cardiovascular disease; ECG, electrocardiogram; HR, hazard ratio; CI, confidence interval

© 2024 Yagi et al. *JAMA Internal Medicine*.
