# Supplementary material for: Routine Electrocardiogram Screening and Cardiovascular Disease Events in Adults
Source: JAMA Intern Med. 2024 Jul 1;184(9):1035–44. doi: 10.1001/jamainternmed.2024.2270 (PMC11217891; doi:10.1001/jamainternmed.2024.2270)
Supplement: Supplement 2. — Data Sharing Statement [file jamainternmed-e242270-s002.pdf]

Data Sharing Statement

Yagi. Routine Electrocardiogram Screening and Cardiovascular Disease Events in Adults.
JAMA Intern Med. Published July 01, 2024. doi:10.1001/jamainternmed.2024.2270

Data
Data available: No

Additional Information
Explanation for why data not available: Data cannot be shared because of the confidentiality contract with the data provider.
